# Actions as perceptual experiments

**DOI:** 10.1167/jov.26.5.10

**Published:** 2026-05-27

**Authors:** Eckart Zimmermann, Antonella Pomè

**Affiliations:** 1 Institute for Experimental Psychology, Heinrich Heine University Düsseldorf, Düsseldorf, Germany

**Keywords:** serial dependencies, saccades, head movements, time

## Abstract

How do we know where (and when) things happen? We review evidence that actions operate as experiments that test and recalibrate internal spatial hypotheses. Postsaccadic errors across ∼100,000 daily saccades induce serial dependencies that shift visual localization. During eye–head gaze shifts, the brain compares expected self-generated motion with sensed motion; subtle velocity-gain perturbations bias perception and head kinematics. In locomotion, manipulating optic-flow speed changes walked distance and, in turn, rescales perceived depth. Temporal reproduction shows serial dependence within action but little transfer to visual time perception. Together, these findings support a unifying perspective on visual space perception as a permanent and active probing of our internal predictions about the environment.

## Sensorimotor serial dependencies

The famous detective Sherlock Holmes once remarked to his companion Watson: “I am a brain; the rest is mere appendix” (Doyle, 1927). Although Holmes tended to speak in a deliberately exaggerated manner, he reflects the prejudice of the brain as a passive receiver of external content and the body as a mere receiver of commands.

Every moment of our lives depends on knowing where we are, where things are, and when events unfold. From catching a ball, to driving a car, to reaching for a cup in the dark, our daily competence relies on remarkably precise judgments of space and time. Yet, these judgments are not simply read off the environment like measurements from a camera. Instead, they are actively constructed and continuously recalibrated through movement. Each glance, head turn, step, or reach generates predictions about the world—and the tiny mismatches between prediction and outcome serve as teaching signals that refine how we see and experience space and time. In this perspective, perception is not a passive registration of reality but an ongoing exchange between action and sensation. By framing actions as perceptual experiments, we offer a unified account of how everyday movements—from the ∼100,000 saccades we make each day to the distances we walk—quietly shape the spatial and temporal structure of our experience. The passive view of perception, in which the visual system would act like a camera, has not been confirmed by empirical data and theories. For example, Rucci, Ahissar, and Burr (2018) argued that visual space is not encoded purely as a static spatial map on the retina; rather, it is actively constructed through temporal processing driven by eye movements. These eye movements transform spatial patterns in the world into temporal modulations of luminance on the retina. The visual system exploits these time-varying signals to encode spatial information. Saccades emphasize low spatial frequencies, and drift enhances high spatial frequencies, leading to a dynamic coarse-to-fine analysis of scenes. In this view, perception is active, as the timing of neural responses, which is shaped by eye movements, is central to representing fine spatial detail.

**Figure 1.**
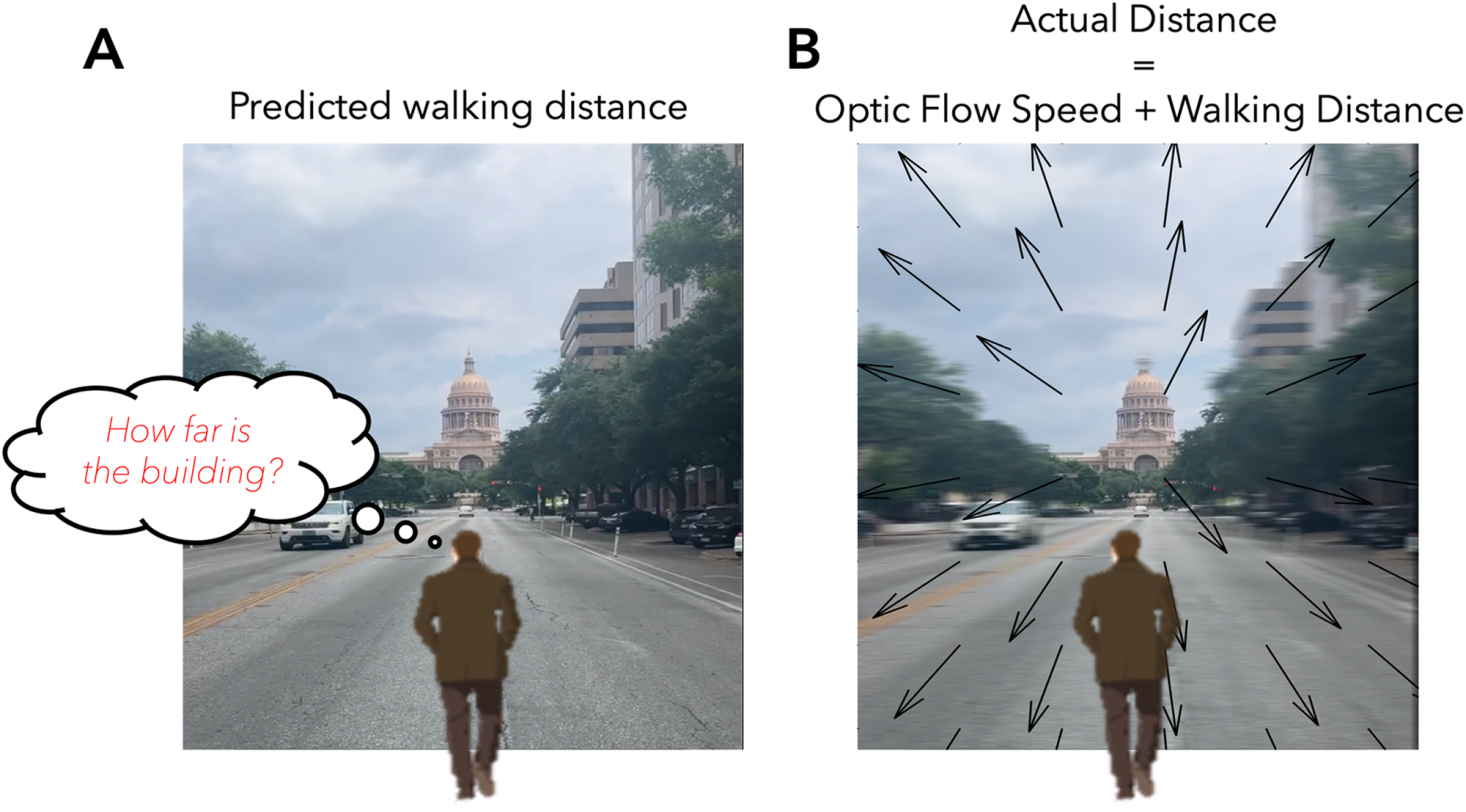
(**A**) Monocular and binocular cues provide information about distance, but how can the brain translate them into the experience of space? By measuring the actual distance when walking to an object, the brain can relate predicted and physical distance. (**B**) During locomotion, internal estimates of traveled distance can be compared with the sensory consequences of movement, including optic flow and body-based signals. The mismatch between predicted and actual sensory input provides an error signal that can update subsequent estimates of visual space.

Hermann von Helmholtz popularized the idea that performing actions can be considered a test of our internal estimates of the external world (Helmholtz, 1948). Consider judging the distance of a building in front of you (Figure 1A). Monocular and binocular cues must be interpreted or scaled in order to be translatable into the experience of spatial distance. We argue that only when linking visual space to motor space can distance be experimentally interpreted. Walking to the building can be considered a measure of space that teaches how to interpret the monocular and binocular cues. After the object has been reached, the actual experienced walking distance might be compared to the predicted one to update or recalibrate future predictions. Actions not only bring the body to a new position but could also be considered as experiments that update our perceptual knowledge about the external world (Figure 1B).

How the true three-dimensional (3D) distances in external space can be inferred from the retinal image has long fascinated researchers in perception (for a review, see Qian, 1997). Although the relative two-dimensional distances are preserved in the retinal projection, the perception of 3D space must rely on additional sources. And, even if the existence of two eyes allows us to reconstruct it, it must be known how this signal scales with external space. Already in 1709, the Irish Bishop Berkeley remarked that only motions of the body can reveal the unknown scale between internal and external space (Berkeley, 1948). A bit more than three centuries later, virtual reality devices provide a convenient means to manipulate the relationship between action and perception when walking in 3D space.

Demonstrating that action influences perception requires experimentally manipulating action planning or movement dynamics and then measuring how those manipulations alter perceptual experience. A classic approach in sensorimotor adaptation experiments is to shift the movement target to a new position while the action is ongoing. The resulting movement error between the final movement position and the new target location will change the planning or dynamics of subsequently performed movements. Sensorimotor adaptation usually is applied for several trials until it reaches a steady state. However, adaptation effects have also been measured on a trial-by-trial basis. If actions are considered a test of perceptual estimates and a means to improve them, then trial-by-trial effects between motor errors and perceptual estimates should exist, because we rarely perform the same action over and over again. We term these sensorimotor serial dependencies, mirroring the attractive biases of current perceptual estimates toward recently experienced stimuli that have been extensively documented in perception (Cicchini, Mikellidou, & Burr, 2024; Manassi & Whitney, 2024). In this perspective article, we review primarily our own recent studies supporting the idea that our perceptual experience of the external world is dynamically shaped by the outcomes of previous sensorimotor interactions. Whether executing rapid eye movements, coordinated head rotations, or traversing our environment, each action serves as a hypothesis test, informing and updating internal predictions.

## Saccades

Visual perception emerges from recurring cycles of gaze shifts and fixations (Kowler, 2011). What is sensed before, during, and after a gaze shift fundamentally contributes to our perception of external space. The execution of a saccade not only serves to bring objects from low to high resolving parts of the receptor array but also provides essential information about external space. The accuracy of a spatial map can only be evaluated by comparing it against reality. Neural maps of space can be calibrated only through performing an action. The ensuing error between the desired and the actual gaze location will reveal the accuracy of the internal map. Gazing around a scene generates a spatial metric, with coordinates mapped onto motor commands. In this view, knowing where something is means knowing how to get there. Although a rough spatial outline is stored as a memorized scene gist, the code for visual localization of objects in the scene might be embedded in the saccade vectors used to foveate the object.

We have explicitly tested the theory of actions as experiments by providing observers with false feedback about their saccades (Cont & Zimmermann, 2021). In the laboratory the saccade target can be displaced after an eye tracker has detected saccade onset. Due to impoverished intrasaccadic displacement detection, subjects do not perceive the jump consciously (Deubel, Schneider, & Bridgeman, 1996). When the eye has landed, the sensorimotor system detects the discrepancy between the intended and the actual postsaccadic eye position. The postsaccadic error will have consequences for subsequently performed saccades. The following saccade is adjusted to correct for the apparent error in saccade execution. Research has shown that repeated systematic exposure to postsaccadic errors leads to gradual adjustments in saccadic amplitude over trials. This phenomenon has been termed *saccadic adaptation* (for a review, see Herman, Blangero, Madelain, Khan, & Harwood, 2013). Postsaccadic errors modify not only saccade amplitudes but also visual localization of objects in space (Zimmermann & Lappe, 2009; Zimmerman & Lappe, 2010)

A recent computational model explains saccadic and visual changes by the modification of three neural signals representing the spatial target percept, the motor command, and the dynamics of a forward model which transforms the efference copy from motor into visuospatial coordinates (Masselink & Lappe, 2021). In this model, a postdictive motor error is calculated by relating the postsaccadic error to the presaccadic target location via the efference copy signal. Postdiction triggers a learning process that likewise affects perception, the motor command, and the efference copy signal.

The functional value of saccade adaptation might be to counteract pathological cases of eye movements. Eye muscle diseases that affect the size of saccades could be corrected by adaptation (Optican, Zee, & Chu, 1985). However, many results show that saccade adaptation has functional relevance far beyond the compensation of muscle fatigue (for a review, see Zimmermann & Lappe, 2016). Postsaccadic errors inform not only about muscle but also about higher-level inaccuracies related to the processing of spatial information. Our lab has reported that modifications in saccade amplitudes can already occur after a single postsaccadic error (Cont & Zimmermann, 2021). Even when intrasaccadic target displacements are unsystematic and vary in size and direction across trials, saccade amplitudes are driven by the postsaccadic error from the preceding trial. Unsystematic postsaccadic errors might be much more frequent in real life. Because postsaccadic errors resulting from noise in the sensorimotor system will have a much higher prevalence than eye muscle diseases, adaptive saccadic amplitude changes might serve a much more general function that is active after every saccade. Postsaccadic errors instruct the metric of motor and visual space (Zimmermann & Lappe, 2016). In this view, performing a saccade probes the validity of the internal mapping of two-dimensional distance.

**Figure 2.**
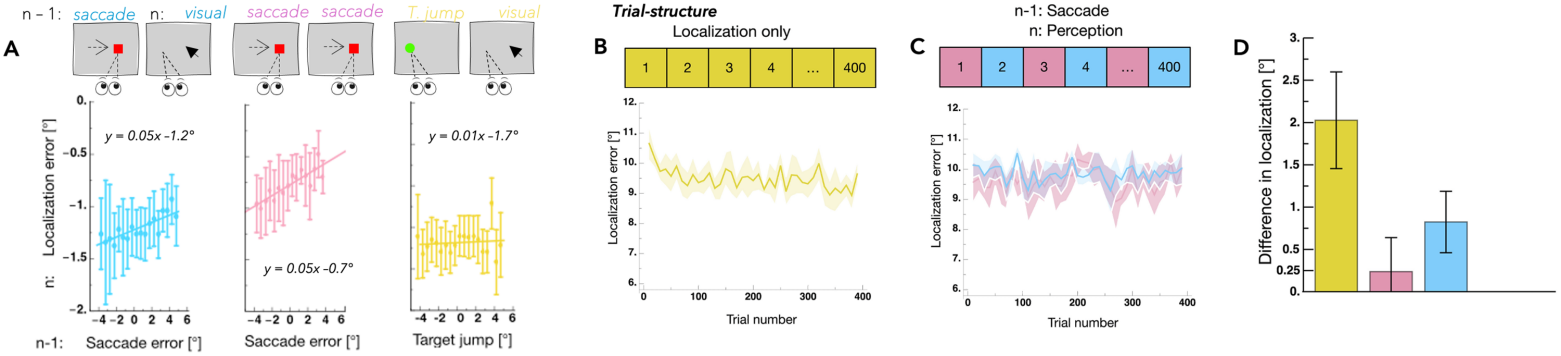
(**A**) Illustration of trial sequences across the three conditions in Experiment 1. Trials *n* – 1 and *n* alternated throughout each experimental session. In Condition 1 (blue), participants fixated on a central point during trial *n* – 1. After 1000 to 1500 ms, the fixation point disappeared and a peripheral target appeared, prompting a rightward saccade. In trial *n*, participants maintained fixation for the entire duration. After 1000 to 1500 ms, a target briefly appeared (300 ms), and participants localized its position using a mouse pointer while maintaining gaze on the fixation point. In Condition 2 (pink), participants executed a saccade toward a target in both trial *n* – 1 and trial *n*, with the target displaced during saccade execution in each case. In Condition 3 (yellow), participants in trial *n* – 1 passively observed a target displacement equivalent in magnitude to that used in Condition 2. Trial *n* was identical to the localization task described in Conditions 1 and 2. (**B**) Visual localization performance in the “localization only” condition, along with the trial-by-trial structure. Localization responses were grouped into bins of 10 trials. The thick line represents the average across all participants, and the shaded area indicates the standard error of the mean (*SEM*). (**C**) Visual localization in the “*n* – 1: saccade, *n*: perception” condition, collected in a separate session. The bar diagram above the graph illustrates the corresponding trial structure. Pink data points represent saccade landing positions in trial *n* – 1, and blue data points show visual localization in trial *n*. (**D**) Mean difference in localization between the “localization only” and “*n* – 1: saccade, *n*: perception” conditions. Color coding follows panels (**B**) and (**C**). *Error bars* denote the *SEM*. Figure is adapted from Cont and Zimmermann (2021).

In our experiments (Figure 2), subjects performed saccades, followed by trials in which they localized a visual target (Cont & Zimmermann, 2021). Localization was assessed under conditions of ocular fixation and in the absence of visual landmarks. We found that visual localization mirrored the dependency observed for saccade amplitudes in response to postsaccadic errors. If, in the previous trial, the postsaccadic error indicated that the saccade was too short to reach the target, then in the current trial observers visually localized the target further into the periphery. Importantly, the execution of a saccade was necessary to induce shifts in visual perception. In a control condition, subjects observed target displacements while keeping ocular fixation. Under this condition, no serial dependencies between the target displacements and localization were found (Figure 2A).

The dependence of perception on actions suggests that preventing observers from performing actions should lead to a deterioration of visuospatial estimates. If visual space perception relies on constant self-monitoring via postsaccadic errors, then localization performance should decline if subjects are forced to maintain prolonged ocular fixation. Indeed, we observed that subjects mislocalized a visual target closer to the fovea than its actual position after prolonged fixation (Figure 2B). When we repeated the experiment, allowing subjects to perform a saccade every second trial (Figure 2C), both visual localization and saccadic targeting were accurate as usual (Figure 2D).

Although adaptive modifications of saccade amplitudes are computed within the cerebellum (Herzfeld, Kojima, Soetedjo, & Shadmehr, 2018), cortical processing of the postsaccadic error has been reported. Neurons in posterior parietal cortex with persistent pre- and postsaccadic responses reflect the intended saccade landing based on efference copy information, whereas neurons with late postsaccadic responses represent the actual saccade ending position (Zhou, Liu, Lu, Wu, & Zhang, 2016). Brain imaging studies in humans suggest that saccade adaptation involves cerebral areas, such as the supplementary eye fields, temporal lobe, and posterior insula (Blurton, Raabe, & Greenlee, 2012); the temporoparietal junction and human middle temporal/V5 complex (hMT/V5), the frontal eye fields (Gerardin, Miquée, Urquizar, & Pélisson, 2012); and the precuneus (Guillaume, Fuller, Srimal, & Curtis, 2018).

**Figure 3.**
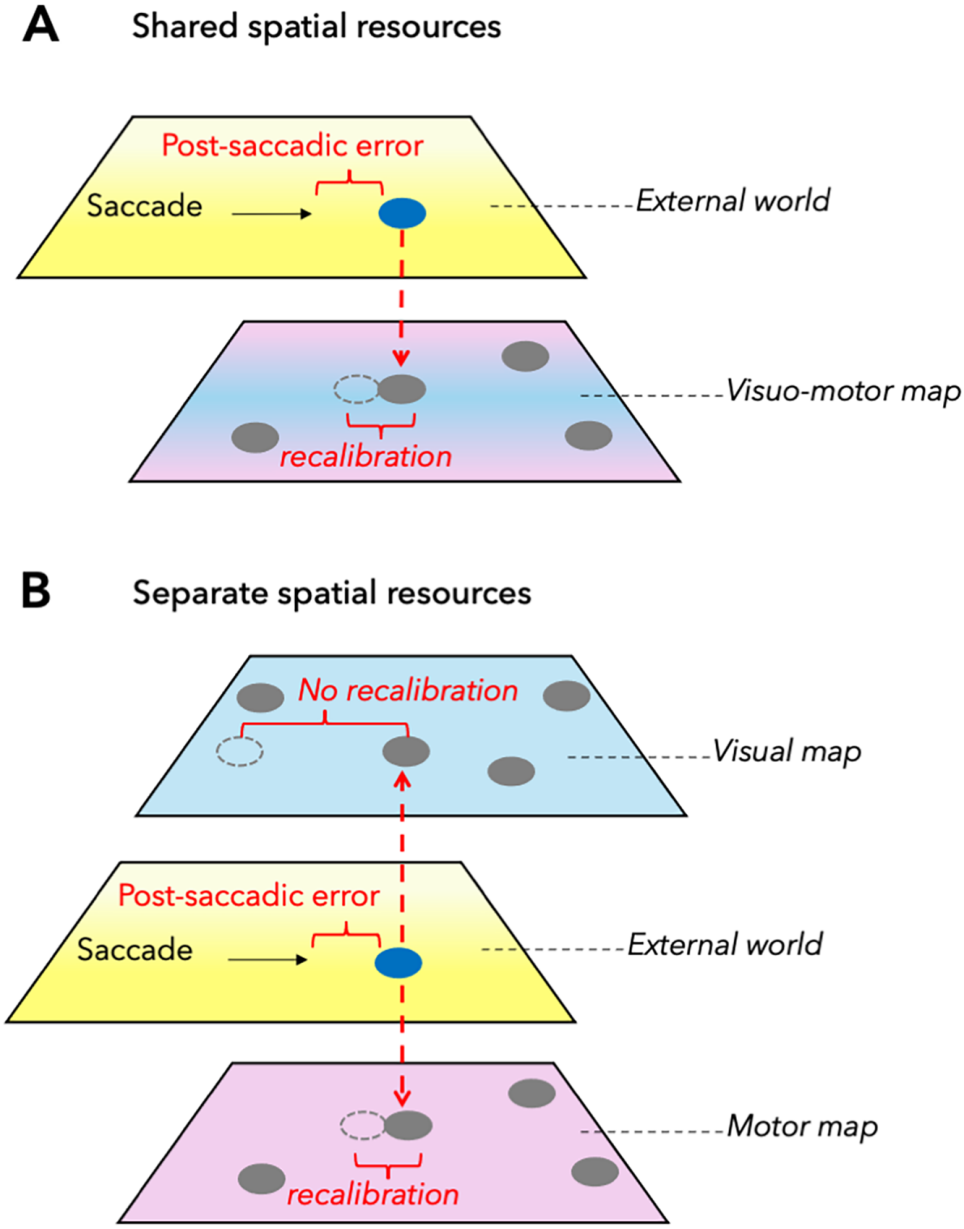
Postsaccadic error as a calibration signal for visual and motor space. (**A**) Saccades to external targets generate postsaccadic errors, defined as the distance between the eye position after saccade landing and the target position. These errors provide a measurable calibration signal that links motor actions to visual space and allows the spatial metric to be updated. Gray disks indicate internal target representations before action, and the dashed circle indicates the recalibrated representation after action. Postsaccadic error signals can recalibrate both perceptual and motor space. If visual and motor space rely on a shared spatial resource, the common coordinates should be updated together, and perception and action remain aligned. Alternatively, if visual and motor space rely on separate resources, (**B**) the same motor error signal can recalibrate each representation independently. In this case, motor adaptation can remain stable while visual localization shifts, revealing dissociable adaptive states despite a common calibration signal. Figure is adapted from Cont and Zimmermann (2021).

The idea that visual space is constructed from motor signals inevitably leads to the question of whether perceptual and motor space rely on a shared resource. In that case, each change in the coordinates of the shared resource would affect perception and action alike, such that they remain constantly aligned (Figure 3A). However, it is also possible that motor error signals affect perceptual and motor space while each is processed separately (Figure 3B). If we could create a situation in which motor and visual space show different adaptive states, this would argue for separate processing. We adapted saccades via intrasaccadic target displacements (Tyralla, Pomè, & Zimmermann, 2023). When the saccades were adapted, we predicted saccade landing online and displaced the target such that the postsaccadic error was effectively annulled. We had previously shown that visual space requires constant updating by motor errors; otherwise, localization shifts toward the fovea. Motor adaptation, however, should remain at a steady-state level under the annulling procedure, as no error signal is registered that would drive further adaptation. We found that, when the number of trials without postsaccadic error was sufficiently large, visual localization shifted toward the fovea. However, in contrast to the idea of a shared resource, motor adaptation remained constant. This dissociation between the adaptation states of saccades and perception strongly suggests that postsaccadic error is used to recalibrate saccades and perception separately. Importantly, both are recalibrated by the same motor error signal.

## Head movements

In natural viewing conditions, eye movements rarely occur in isolation. Even when seated, head movements typically accompany ocular movements. Under this condition, calibrating visual space through actions becomes more difficult, as the sensorimotor system must consider its own contribution to the visual motion experience. Moving the visual receptors across the scene produces a motion stimulus on the retina that must not be confused with motion from the external world. When we move our eyes, the self-produced visual motion is omitted; however, when we move our head, we see visual motion that usually does not appear as external motion to us (Hadani & Julesz,1994). The sensorimotor system therefore compares the sensed with the expected visual motion. Many studies have highlighted that perceptual stability is not a passive process; instead, it is an active, learned interpretation of changing sensory input, combining visual information with signals about our own movements (Jaekl, Jenkin, & Harris, 2005; Wallach, 1987; Wertheim, 1994; Wexler, 2003).

To determine the velocity of the head movement, humans rely on three sources: vestibular signals, which report the position and movement of the head in space; neck proprioception, representing a head-on-trunk signal; and an efference copy, consisting of a copy of the motor command (Crowell, Banks, Shenoy, & Andersen, 1998; Mergner, Rottler, Kimmig, & Becker, 1992). An efference copy encoding the intended displacement vector of head movement could, in theory, be used to predict the speed of visual scene displacement. However, its utility is constrained by potential variability in the state of the neck muscles (Monjo, Terrier, & Forestier, 2015; Scotland, Adamo, & Martin, 2014) and by the possibility of ongoing adjustments to head movement control during execution (Zimmermann, 2021).

**Figure 4.**
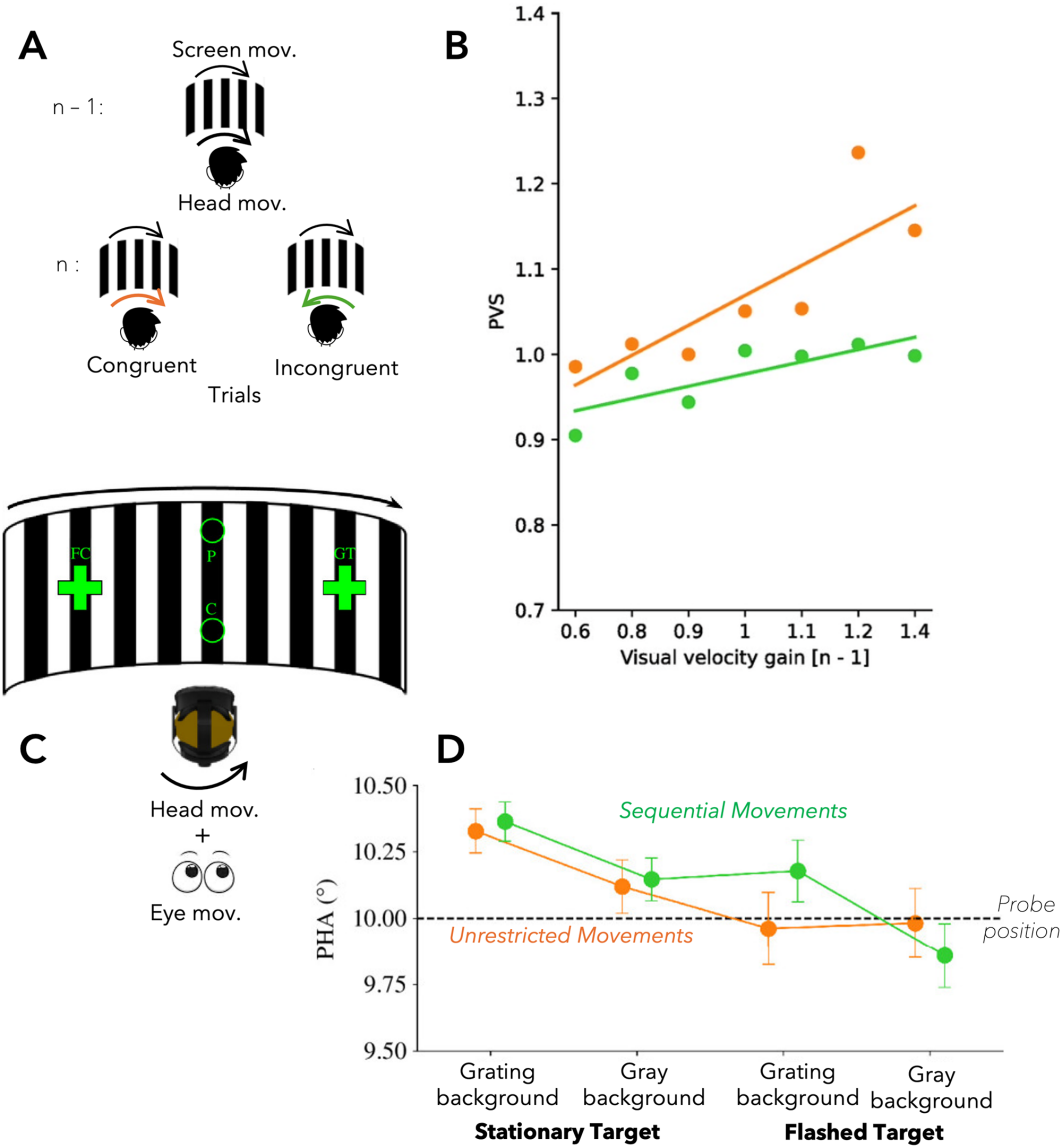
(**A**) Serial dependence combinations. This illustration depicts various combinations of visual velocity gain and head movement direction. Orange arrows represent the direction of head movement, and blue arrows indicate the perceived relative motion of the visual environment. For example, when a visual velocity gain greater than unity is applied, the visual scene moves more rapidly across the retina compared to a condition with unity gain. (**B**) Slopes of the point of visual stability (PVS) in trial *n* as a function of head movement congruency in trial *n* – 1. PVS values are shown as a function of visual velocity gain in trial *n* – 1 for an example participant, separately for trial pairs with congruent and incongruent head movements. Data are fitted by a linear function (fit function for congruent trial pairs: *y* = 0.77 + 0.30*x*; fit function for incongruent trial pairs: *y* = 0.85 + 0.12*x*). A clear linear relationship can be observed in the congruent condition, indicating a stronger influence of the prior visual velocity gain on the current PVS. In contrast, the relationship is notably weaker in the incongruent condition, suggesting reduced serial dependence. (**C**) Illustration of the experimental setup: Participants executed either eye-only or combined eye–head gaze shifts from a central fixation cross (FC) to a gaze shift target (GT). Following the gaze shift, they were required to judge the position of a comparison stimulus (**C**), presented afterward, relative to a probe stimulus (P), which was shown prior to the gaze shift. Notably, the grating pattern depicted in the illustration was visible only during the gaze shift and was not present during the presentation of the stimuli. (**D**) Point of horizontal alignment (PHA) in degrees for the stationary targets and flashed targets during both grating and gray background. The dashed line represents the position of the probe (PP). Color-coded for restricted and unrestricted movements. Figure is adapted from (Bayer & Zimmermann, 2023; Bayer & Zimmermann, 2024).

For example, if the visual scene is artificially altered to move faster or slower during head movements, observers easily detect the external motion, often resulting in motion sickness (Wiesing, Zimmermann, Schmidt, & Buchner, 2024). We wondered how the brain establishes the link between a certain head movement velocity and the corresponding visual motion (Bayer & Zimmermann, 2023). In a head-mounted display (Figure 4A), we showed observers a patterned background that moved while the subjects rotated their heads. On every trial, the velocity gain of the visual movement was set slightly lower or higher than the speed of the head movements. Subjects were required to respond whether the background moved faster or slower than their head movement. We found that estimates of normal visual motion speed depended on the visual motion seen in the previous trial. If in the previous trial the visual motion was slightly faster than the head movement speed, subjects accepted a faster visual motion in the current trial as normal and vice versa (Figure 4B). These data indicate that what we accept as the sensory consequences of our own actions depends on previous experiences when interacting with the environment.

Head movements, like saccades, can be used to measure the distance of objects in space. Head movements, which last on average between 400 and 800 ms (Andres & Hartung, 1989; Hoffmann, Chan, & Heung, 2017), enlarge the field of vision, and targets with an eccentricity exceeding 20° automatically trigger a combined eye–head gaze shift (Zangemeister & Stark, 1981). To understand how head movement signals interact with spatial processing, we implement a spatial updating task, which has been extensively studied in eye movements (Golomb & Mazer, 2021). An eye–head gaze shift starts with a saccade to the target location, followed by the head movement. During the head movement the vestibule–ocular reflex rotates the eyes in the opposite direction of the head movement to stabilize the image in the retina. When the head moves, relative retinal motion remains, because objects at the fixation distance remain roughly stable; nearer objects move opposite the head direction on the retina, and farther objects move with the head direction on the retina.

We asked subjects to visually localize targets either across a saccade while the head remained still or across an eye–head gaze shift (Figure 4C). The target was shown before the execution of the respective gaze shift, and subjects compared the position of the target with a comparison stimulus after gaze shift performance. We found that the accuracy of visual localization was higher after participants performed an eye–head gaze shift. Visual motion produced by the head movement affects visual localization. When we artificially increased the visual motion speed, subjects overestimated the target position (Figure 4D). Visual motion experienced during the eye–head gaze shift might contribute to measuring the spatial distance that the head movement covers. A comparable recalibration has also been reported for smooth pursuit eye movements (Haarmeier, Bunjes, Lindner, Berret, & Thier, 2001; Luna, Serrano-Pedraza, Gegenfurtner, Schütz, & Souto, 2021). Manipulating the speed of the background motion while the eye followed a target showed that the brain adapts to it and recalibrates the prediction of self-generated motion speed accordingly.

## Walking

Only human locomotion consists of full striding bipedalism, characterized by fully extending the hip and knee joints during the stance phase requiring an oscillatory regulation of muscle activity to maintain body posture (McGowan, Neptune, Clark, & Kautz, 2010; Zajac, Neptune, & Kautz, 2002). The visual contingency of locomotion is optic flow; a pattern of radial expansion whose focus indicates heading direction. Gibson (1950) proposed that optic flow patterns may specify self-motion direction and, under conditions of pure translation and available scale information, locomotion speed.

Experiments with stationary observers revealed that optic flow signals are principally sufficient to allow estimates of traveled distance (Frenz & Lappe, 2005; Redlick, Jenkin, & Harris, 2001). During walking, the brain continuously compares predicted outcomes with actual sensory input to guide behavior. Several signals could potentially measure traveled distance, including time traveled in combination with movement speed, step number in combination with step size, or accumulated optic flow. Idiothetic signals—information generated by self-motion, such as proprioceptive feedback, visual cues, or efference copies of motor commands—modulate activity in the primary visual cortex (V1) as well as the perception of self-location within the environment (Niell & Stryker, 2008). Furthermore, active navigation has been shown to influence the amplitude of neural responses along the visual pathway (Diamanti, et al., 2021). In rodents, studies have shown that activity in the primary visual cortex is correlated with hippocampal activity, even when visual feedback is absent. This V1 activity appears to be modulated by the physical distance traveled, suggesting that it may convey top–down predictive signals related to the spatial environment (Fiser, et al., 2016). In humans, locomotion also affects visual contrast perception (Benjamin, Wailes-Newson, Ma-Wyatt, Baker, & Wade, 2018; Cao & Händel, 2019; Davidson, Verstraten, & Alais, 2024, Szekely & MacNeilage, 2025). Internal signals coding traveled distance are thus potentially available to be compared to the perceived visual distance, and the resulting error could drive recalibration of internal visuospatial estimates.

**Figure 5.**
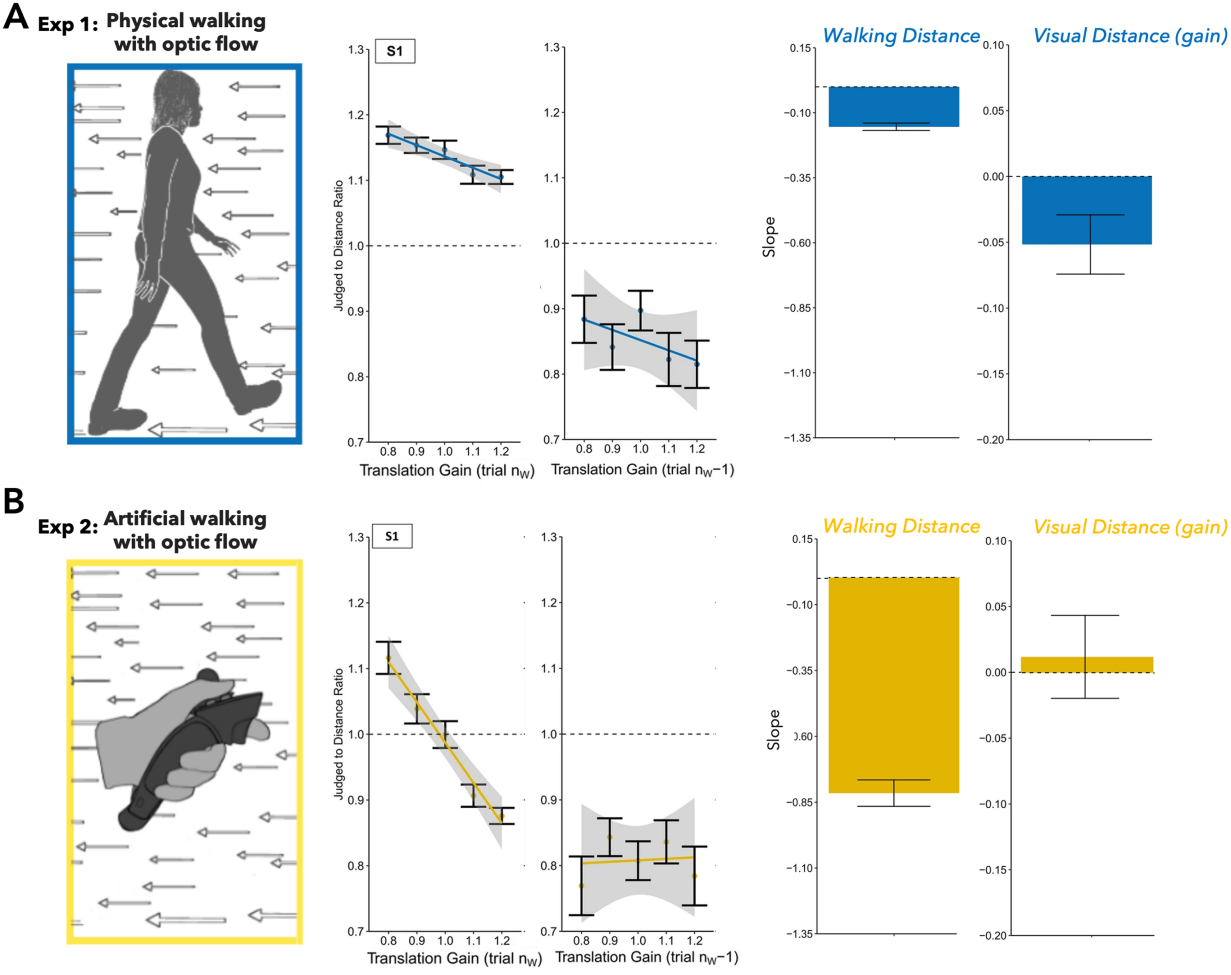
(**A**) Physical walking with optic flow for an example participant. Left panel: Regression analysis between the translation gain in trial *n_W_* and the judged-to-actual distance ratio for walking distance estimations in the same trial. Right panel: Serial dependence effects quantified by a regression between the translation gain in trial *n_W_* − 1 and the visual distance judgments in trial *n_W_*. (**B**) Artificial walking with optic flow for an example participant. Left panel: Regression between the translation gain in trial *n_W_* and the judged-to-actual distance ratio for walking distance estimates within the same trial. Right panel: Serial dependencies quantified by the regression between the translation gain in trial *n_W_* − 1 and the visual distance judgments in trial *n_W_*. Figure is adapted from Wiesing and Zimmermann (2023).

A discrepancy between visual motion and actual bodily movement has been shown to systematically influence later distance judgments in blind-walking tasks (Mohler, Creem-Regehr, & Thompson, 2006; Rieser, Pick, Ashmead, & Garing, 1995). For example, Rieser et al. (1995) altered optic flow by placing participants on a treadmill mounted on a trailer that was pulled by a tractor. This setup allowed the researchers to dissociate visual motion speed (determined by the tractor's movement) from physical walking speed (determined by the treadmill). After adapting to conditions in which visual motion was slower than their actual movement, participants tended to overshoot the target during subsequent blind-walking trials. Conversely, when visual motion was faster than physical movement during adaptation, participants undershot the target location. We showed participants a distance of 2.5 meters in a virtual reality environment (Wiesing & Zimmermann, 2023) (Figure 5). They had to walk either physically (Figure 5A) or virtually (Figure 5B) until they were convinced they had reached the memorized goal of 2.5 meters. While they walked, we either increased or decreased the optic flow speed of the virtual environment. These changes were too small for conscious detection; however, their walking distance increased when optic flow speed decreased and vice versa, as has been observed in previous studies (Mohler et al., 2006; Prokop, Schubert, & Berger, 1997). We predicted that, if body motion measures distances in external space and, through our manipulation, it appears to subjects that they will have to walk farther than usual, then they will update their internal map of perceived 3D space. To test this prediction, in the trial immediately following the walking, subjects localized visual targets verbally in 3D space. If they walked a larger distance in the previous trial, they localized the visual target farther in depth in the current trial, whereas they judged it closer to the body when they walked a smaller distance (see Figure 5, right panels).

## Time

All arguments discussed so far have referred to the perception of space, inevitably leading to the question of whether the same holds true for the perception of time. Precisely timed actions require a tight alignment between the timing of external events on the one hand with their perceptual representation and the respective motor plan on the other. Particularly, when moving to reach a seen object, such as when a predator catches prey, motor timing must be perfectly aligned to the interpretation of visual motion.

Encoding a temporal interval with motor signals of movements that will be initiated to reproduce that interval ensures perfect alignment between perception and action regarding time. Indeed, single-cell recordings in monkeys revealed that temporal intervals are measured prospectively in relation to the desired motor plan to reproduce that interval (Jazayeri & Shadlen, 2015). Furthermore, analogous to spatial recalibration errors of movement in time, responses that were made too early or too late could recalibrate the internal representation of time. History biases on temporal judgments (Jazayeri & Shadlen, 2010; Zimmermann & Cicchini, 2020) also occur on a trial-by-trial level.

**Figure 6.**
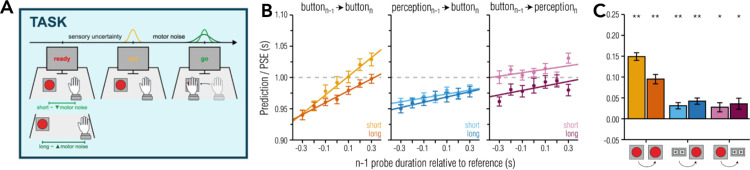
(**A**) Overview of experimental task. In the button task, participants were asked to predict the occurrence of the third stimulus and were told that the interval between the first and second stimulus was predictive of the occurrence of the third stimulus. Upon button press, a red circle was presented on the screen, indicating whether the button press was earlier than, in time with, or later than the third stimulus. We thus created artificial errors with respect to participants’ predictions, and the predicted occurrence of the third stimulus was 1 second in each and every trial. (**B**) Prediction and comparison performance plotted as a function of the previous probe duration (trial *n* – 1) relative to the 1-second reference duration across different task order conditions (yellow/orange: button n – 1/button *n*; blue, perception *n* – 1/button *n*; purple: button *n* – 1/perception *n*) and button distance conditions (light, short; dark, long). Slope and intercept values were derived from linear regressions performed individually for each participant and condition. (**C**) Group-level summary of slope values, indicating serial dependence effects from trial *n* – 1. Figure is adapted from Schlichting et al. (2023).

Several studies have found attractive serial dependencies when subjects had to discriminate or reproduce temporal intervals (Cheng, Chen, Yang, & Shi, 2024; Glasauer & Shi, 2022; Togoli, Fedele, Fornaciai, & Bueti, 2021; Wehrman, Sanders, & Wearden, 2023; Wiener, Thompson, & Coslett, 2014). We aimed to test the idea of perceptual recalibration through actions for temporal events by providing an error signal in the time reproduction task (Figure 6). Subjects were required to perform the ready–set–go task and received false feedback about their performance (Schlichting, Fritz, & Zimmermann, 2023) (Figure 6A). Participants saw two Gabor stimuli that marked an interval of 1000 ms. They had to press a button when a third Gabor should appear such that the interval between the second and the third Gabor matched that between the first and the second. The third Gabor was presented randomly across trials between 700 and 1300 ms. This range of durations was chosen to produce artificial timing errors. For example, if the subject reproduced the first interval more or less correctly after 1000 ms and the third Gabor would appear at 1300 ms, the resulting false feedback would signal that the movement was 300 ms too early. We were then interested if movement timing in the trials immediately following would reflect the false feedback. We found serial dependencies between the false feedback and the moment timing in the next trial. If participants experienced being too early in trial *n* – 1, they arrived later at the button in trial *n* and vice versa for experiencing being too late (Figure 6B).

We wanted to know if motor uncertainty affected the strength of serial dependencies. We manipulated motor uncertainty by letting participants perform short or long movements to reach the button. Long movements resulted in significantly higher movement timing variability than short movements. We found that serial dependencies were stronger when short movements were performed (Figure 6C). This finding shows that the sensorimotor system monitors its own variability and takes it into account when judging the fidelity of the motor error. Errors of less variable movements contain less self-produced motor noise and thus are weighed higher by the sensorimotor system in order to change upcoming movements. The effect of motor variability generalized to different movement types (i.e., foot movements or simple key presses). However, it did not generalize to perceptual time estimates. These data suggest that the recalibration signal modifies timing in a global movement plan upstream of concrete effector control. Such a signal might be necessary to coordinate the start of separate movements.

When individuals intentionally perform an action that produces an outcome (e.g., pressing a button that triggers a tone), they perceive the action as occurring later and the outcome as occurring earlier than they actually do, effectively binding the two events together in time. This phenomenon is widely interpreted as an implicit measure of the sense of agency, reflecting the predictive integration of motor commands by the brain and their expected sensory effects (Haggard, Clark, & Kalogeras, 2002; Moore & Obhi, 2012).

Using a virtual reality paradigm, Suzuki, Lush, Seth, and Roseboom (2019) found identical time compression results in an intentional button press task compared with a passive observation. Based on their results, they argued that intentional-binding-like effects are most simply accounted for by multisensory causal binding, irrespective of whether the button has been pressed actively or passively. However, we replicated their study in virtual reality but failed to reproduce their results (Wiesing & Zimmermann, 2024). We found consistently that in active conditions subjects estimated the temporal intervals as significantly shorter as in the passive condition.

## Discussion

We have reviewed studies that support the idea that actions test and update perceptual hypotheses. We focused on visual perception, which defines the goals for almost all our actions. For gaze shifts, we found strong dependencies between movement errors and changes in spatial perception. Saccades are the movements most well suited to play the role of an experimenter that instructs internal spatial maps. We perform about 100,000 per day, and their errors are precisely defined. Our need to move is not an ancillary function but rather a fundamental aspect of maintaining and enhancing perceptual accuracy. The motility of the eye transcends mere compensation for the inhomogeneous distribution of photoreceptors across the retina; it serves as an essential mechanism for actively testing, refining, and updating our internal models of the environment. Through the oscillatory nature of gaze shifts and ocular fixation, we engage in perceptual hypothesis testing that allows us to continuously adapt our understanding of our surroundings. Eye movements facilitate the generation of predictions regarding the state of the environment, even in areas that are not currently within our central field of vision.

Serial dependencies also occur between walking distances and localization of objects in depth. We found that manipulations of the optic flow during locomotion changed the walking distances and affected visual space. It is not yet clear whether the manipulation of the self-generated optic flow directly influenced visual estimates or whether the effect was mediated via the change in walking distance. The perspective of actions as perceptual experiments is echoed in contemporary research demonstrating that visual estimates are heavily influenced by recent sensory experiences and the biases introduced through serial dependencies. Our ability to localize objects and discern their features is not solely reliant on retinal input but is significantly enhanced by our sensorimotor experiences, which allow us to recalibrate our internal spatial maps through active engagement with the environment.
